# The dynamics of electric powered wheelchair sideways tips and falls: experimental and computational analysis of impact forces and injury

**DOI:** 10.1186/s12984-016-0128-7

**Published:** 2016-03-02

**Authors:** Brett Erickson, Masih A. Hosseini, Parry Singh Mudhar, Maryam Soleimani, Arina Aboonabi, Siamak Arzanpour, Carolyn J. Sparrey

**Affiliations:** School of Mechatronic Systems Engineering, Simon Fraser University, 250-13450 102 Ave., Surrey, BC V3T 0A3 Canada; MobiSafe Systems Inc., Room 5330 250-13450 102 Avenue, Surrey, BC V3T 0A3 Canada; International Collaboration on Repair Discoveries (ICORD), Vancouver Coastal Health Research Institute and The University of British Columbia, Vancouver, BC Canada

**Keywords:** Mobility devices, Simulation, Optical tracking, Dummy, Rigid body dynamics, Head injury criteria, Seatbelt

## Abstract

**Background:**

To reduce the occurrence of wheelchair falls and to develop effective protection systems, we aimed to quantify sideways tip and fall dynamics of electric power wheelchairs (EPWs). We hypothesized that driving speed, curb height and angle of approach would affect impact forces and head injury risk for wheelchair riders. We further expected that fall dynamics and head injury risk would be greater for unrestrained riders compared to restrained riders.

**Methods:**

Sideways wheelchair tip and fall dynamics were reconstructed using a remotely operated rear wheel drive EPW and a Hybrid III test dummy driving at different approach angles (5 to 63°) over an adjustable height curb (0.30 to 0.41 m) at speeds of 0.6–1.5 m/s. Rigid body dynamics models (Madymo, TASS International, Livonia, MI) were developed in parallel with the experiments to systematically study and quantify the impact forces and the sideways tip or fall of an EPW user in different driving conditions.

**Results:**

Shallower approach angles (25°) (*p* < 0.05) and higher curbs (0.4 m) (*p* < 0.05) were the most significant predictors of tipping for restrained passengers. Unrestrained passengers were most affected by higher curbs (0.4 m) (*p* < 0.005) and fell forward from the upright wheelchair when the approach angle was 60°. Head impact forces were greater in unrestrained users (6181 ± 2372 N) than restrained users (1336 ± 827 N) (*p* = 0.00053). Unrestrained users had significantly greater head impact severities than restrained users (HIC = 610 ± 634 vs HIC = 29 ± 38, *p* = 0.00013) and several tip events resulted in HICs > 1000 (severe head injury) in unrestrained users.

**Conclusions:**

Sideways tips and forward falls from wheelchairs were most sensitive to curb height and approach angle but were not affected by driving speed. Sideways tips and falls resulted in impact forces that could result in concussions or traumatic brain injury and require injury prevention strategies. Seat belts eliminated the risk of falling from an upright chair and reduced head impact forces in sideways wheelchair tips in this study; however, their use must be considered within the ethical and legal definitions of restraints.

## Background

As with all mobility devices, wheelchairs are subject to events that can lead to passenger injury. In one study, over 87 % of wheelchair users reported at least one tip or fall in the past three years [1]. Approximately 10 % of wheelchair users are driving electric powered wheelchairs [2]. Electric power wheelchairs are designed for stability and therefore rarely tip going over low height obstacles [3]. EPWs are more likely to tip sideways and those sideways tips or falls are more likely to require medical attention [4]. Traumatic brain injuries and concussions are among the most common injuries requiring hospitalization resulting from wheelchair falls [5]. Riders not restrained in their chairs may fall from the chair when the chair stops or jolts unexpectedly even though the chair remains upright. A previous experimental study using an EPW and a crash dummy found no significant correlation between driving speed and tips or falls for a limited number of real world environments; however, falls had a significant inverse correlation with seat belt use [3]. To reduce the occurrence of wheelchair falls and develop effective protection systems, we must quantify the tip and fall limitations of EPW’s and determine the impact severity and potential consequences for riders.

Computational models are powerful tools for systematically studying fall dynamics and evaluating the effects of specific fall parameters on impact forces and injury. Rigid body dynamic analyses were originally developed to study motor vehicle impacts [6]. However, the same computational tools are now used to simulate falls [7–9]. Lower intensity backwards falls from standing were accurately modeled with rigid body simulations and the impact energy and post impact kinematics correlated with experimental falls [8]. Parametric rigid body simulation of falls from playground equipment quantified the effect of impact surface characteristics on injury severity [10]. Combined, these previous studies demonstrate the suitability of rigid body dynamics for studying falls in general. However, systematically comparing computational models with controlled experiments is necessary to validate rigid body dynamic simulations for studying wheelchair fall dynamics.

The overall goal of this study was to map the effect of EPW driving variables on fall risk, head injury risk and impact locations for each tip or forward fall. We hypothesized that driving speed, curb height and angle of approach would affect impact forces and head injury risk for wheelchair riders. We further expected that the impact forces and head injury risk would be greater for unrestrained riders compared to restrained riders. Quantifying the impact forces and injury risk associated with power wheelchair falls is important for identifying opportunities to reduce falls and to establish the design criteria for effective injury prevention technologies when falls occur.

## Methods

### Rigid body dynamic models

Rigid body dynamic analysis models were developed in MADYMO (TASS International, Livonia, MI) to systematically explore the effects of wheelchair speed, orientation and curb height on tip or fall risk, head injury risk, impact force, and impact location for power wheelchair users. Due to the complex design and varied materials used in the powered wheelchair (Express, Ranger Wheelchairs Ltd. Surrey, BC), assigning a single mass and determining inertial characteristics were not feasible. In addition, modifications to the wheelchair, such as changing battery size or moving footrests affect the overall inertia of the chair. Therefore, individual components of the power wheelchair were measured and weighed in triplicate. Inertia for each component was calculated using the average measurements and assuming uniform mass distribution in each part. To construct the wheelchair model, the components were modeled individually and assembled in MADYMO to match the dimensions of the existing chair. Each component was assigned a mass, center of gravity, inertias (I_xx_, I_yy_, I_zz_, I_xy_, I_yz_, I_xz_) and dimensions. MADYMO rigid body models are constructed with cylinders, ellipsoids and plates; therefore each component was approximated with a cylinder, cuboid, ellipsoid or plane. The assembled chair model center of gravity and inertial characteristics were experimentally validated.

The assembled wheelchair model was compared with the physical characteristics of an empty EPW to validate the rigid body model behavior. The center of gravity of the chair was determined using calibrated scales under each wheel as well as assessing the fore/aft and side/side tipping points of the chair. The center of gravity of the modeled wheelchair was validated by comparing the tip angle of the chair in the model with tip angles of the physical wheelchair measured with optical tracking (Oqus Camera System, Qualisys Inc. Goteborg, Sweden). Inertia was validated by comparing the righting dynamics of the simulated wheelchair with the optical tracking position data obtained from physical testing of the wheelchair released from a tipped position.

To study the impact forces on the rider resulting from a wheelchair fall, a calibrated, 50th percentile Hybrid III dummy model was used. The Hybrid III model was selected to match the experimental test configuration and because the Hybrid III model provides more realistic head and neck characteristics than the ISO test dummy often used for wheelchair stability testing. Madymo includes calibrated and validated dummy models to study the effects of external mechanics on load transfer and injury risk in the body. Anthropometry, joint stiffness characteristics and tissue compliance have all been individually validated during model development [11]. Although these models are not explicitly validated for fall simulations, they have demonstrated validity in simulating a variety of fall scenarios [7–10].

To test the effects of passenger restraints on tip and fall dynamics, seat belts were constructed and fit over the dummy model using a belt fitting algorithm that defined belt contact [11], belt material characteristics [12], anchor points and pre-tension. The seat belt was assumed to be properly positioned for all riders, crossing the lap below the iliac crests. The belt was modeled as a finite element web to define contact with the dummy. Cable elements of variable length were assigned to the end of each belt to eliminate slack in the belt while the dummy position equilibrated in the first step of the simulation.

Contact characteristics were defined for the curb and floor assuming a material stiffness similar to concrete [13]. The coefficient of friction between the dummy and the wheelchair was varied between 0.1 and 1.2 at the seat and 0.3 to 1.2 at the footrest to determine the properties which best correlated with experimental results. Bilinear stiffness characteristics (12.5 N/mm up to 100 mm and 125 N/mm above 100 mm) for the wheelchair seat and seat back were approximated from the neoprene wrapped, contoured foam cushions provided with the electric power wheelchair.

### Experimental testing

**Fig. 1:**
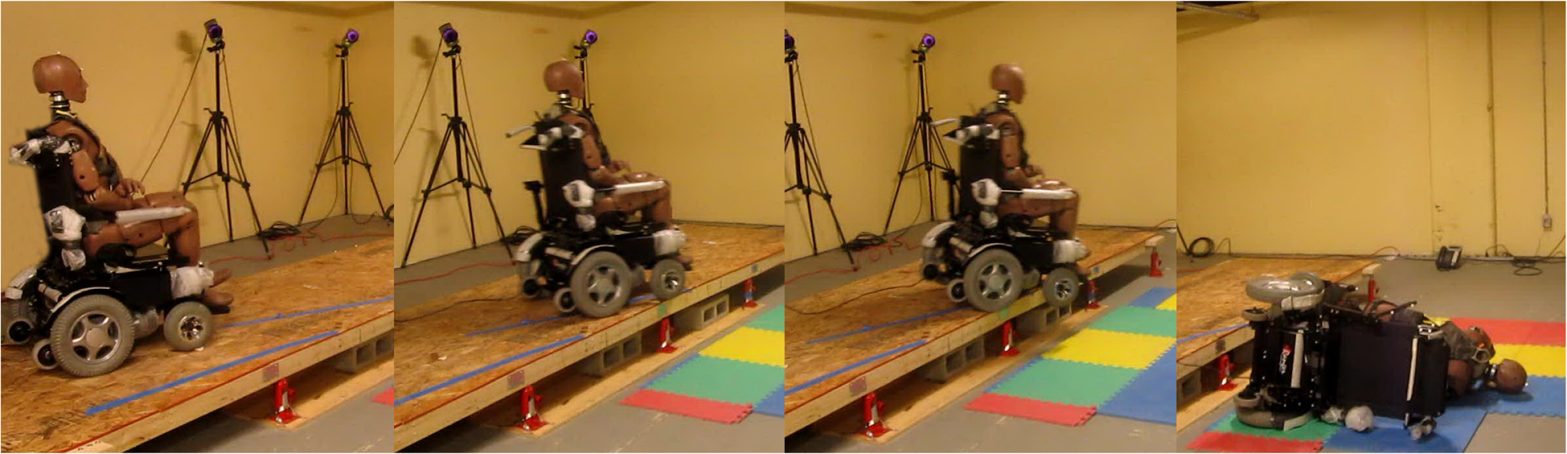
The experimental set up using the rear-wheel drive power wheelchair driven by a remote joystick showing an example of a tip event. The Hybrid III dummy was positioned in the chair and was either unrestrained or belted with a lap belt. The curb height was adjusted using manual jacks under the platform. Lines on the platform guided the operator to different approach angles. The dynamics of each experiment were captured using eight optical tracking cameras mounted around the test structure using reflective markers attached to the dummy and the wheelchair

To assess the validity of the wheelchair fall simulations constructed in MADYMO, a series of experiments were conducted using the same power wheelchair and a 50th percentile Hybrid III dummy (Fig. 1). An adjustable height curb was constructed with angled paths marked on the surface to drive the wheelchair off the curb at a range of orientations (15–60°). A rear-wheel drive power wheelchair (Express, Ranger Wheelchairs Ltd. Surrey, BC) was modified to be driven by wired, remote joystick, away from the fall area. The wheelchair had 12 in. (0.3048 m) drive wheels, 8 in. (0.2032 m) steerable casters and 4 in. anti-tipper wheels on the back. The fully equipped chair weighed 293 lb (133 kg) and the Hybrid III dummy weighed 171 lbs (77.7 kg). Two sets of tests were run: 1) with an empty power wheelchair - to further validate chair and environmental variables and 2) with the dummy positioned in the wheelchair - to validate the complete simulation. The dummy and the wheelchair were instrumented with reflective markers to track the position of the wheelchair frame and dummy head using optical tracking (Oqus Camera System, Qualisys Inc., Göteborg, Sweden). The speed and orientation of the wheelchair varied slightly throughout each test due to the use of the joystick. The actual speed and orientation of the wheelchair immediately before going over the curb were determined from the motion capture data. In total 6 empty-chair trials and 8 occupied, non-restrained trials were completed with speeds ranging from 0.6 to 1.5 m/s, orientation from 5 to 63° (where zero degrees is parallel and 90° is perpendicular to the curb) and curb height from 0.30 to 0.41 m. Each test was reconstructed in MADYMO for validation. Landmarks from the motion capture study were input into the simulations to compare the simulated fall studies with the experimental results.

### Sensitivity analysis of wheelchair driving parameters

**Fig. 2:**
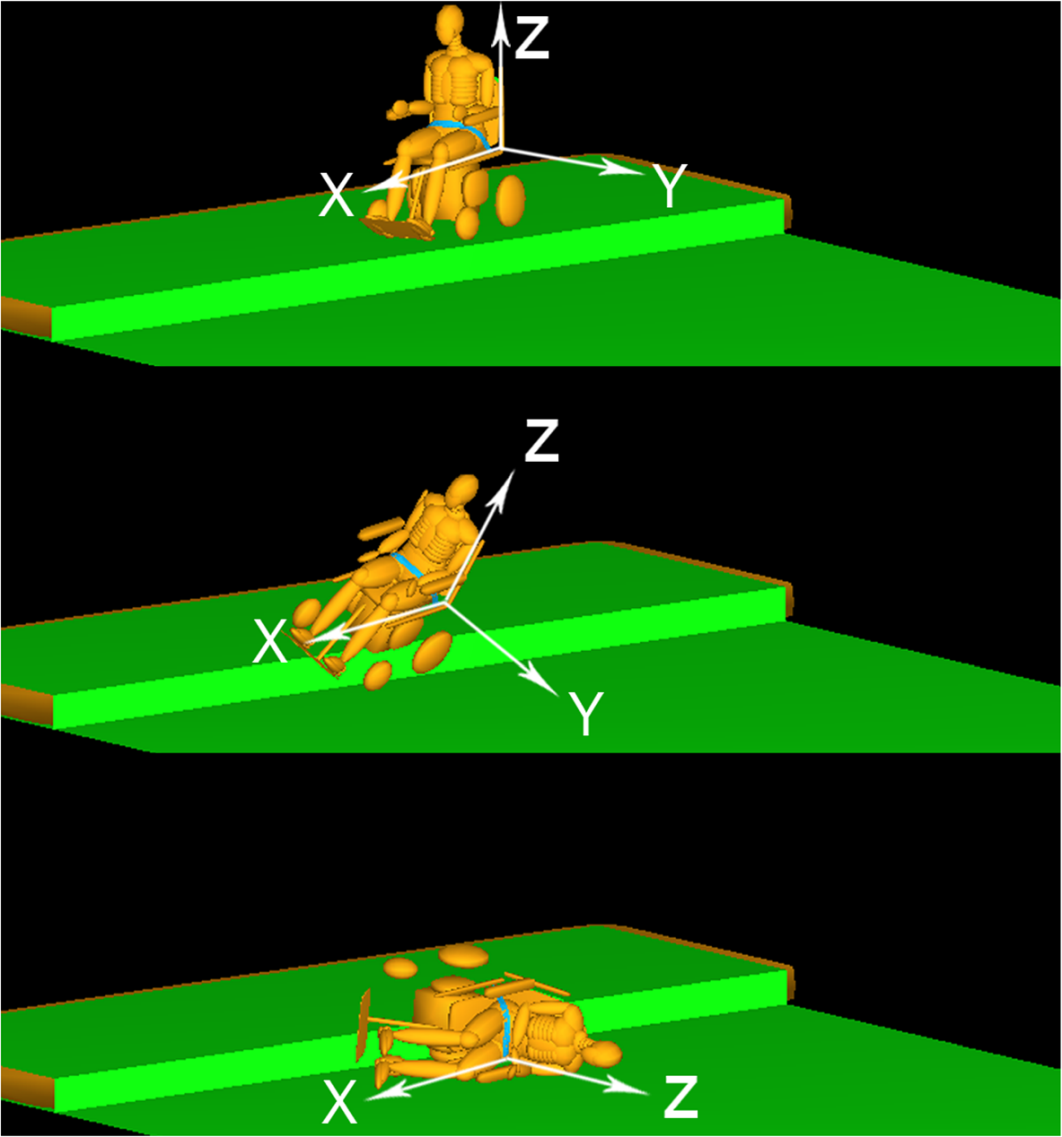
Example MADYMO power wheelchair model showing local coordinate system. The origin of the coordinate system was used as the landmark to describe head impact location relative to the wheelchair after a tip or fall

After validating the model, the effects of environmental variables on wheelchair fall dynamics were systematically studied. Vehicle speed (1 mph (0.4470 m/s), 2 mph (0.8941 m/s), or 3 mph (1.3411 m/s)), incident angle (25, 40 or 60°), curb height (0.2, 0.3 or 0.4 m), and seat belt use (yes/no) were simultaneously altered to generate 54 simulations of wheelchair falls. The impact location relative to the wheelchair reference frame (Fig. 2), head impact force and head injury criterion (HIC) were recorded for each simulation.

To determine the factors with the greatest influence on injury severity during a wheelchair fall, statistical analyses quantified the effects of driving parameters on impact location and HIC. The 15 ms HIC was used to determine head injury risk [14]. The kinematics of wheelchair tips are not the same as those of rider falls, where the wheelchair remains upright; therefore, dynamics of wheelchair tips were analyzed separately from rider falls. Due to the near instantaneous nature of all impacts, head position at impact was determined by averaging three time points of position data centered on the time correlating with peak force. Using linear regression (Matlab R2014, Mathworks Inc, Natick, MA), the effect of driving parameters (speed, orientation and curb height) on HIC values were tested, as well as the correlation with head position. Significance was assumed when *p < 0.05*. Multinomial logit regression was used to determine the correlation between driving parameters and tip or fall risk. The effect of seat belts on head injury risk was assessed using a Wilcoxon signed rank test comparing the results of restrained and unrestrained simulations with the same parameters. To eliminate the significant effect of forward falls (when the rider falls but the chair stays upright) on HIC value, a second Wilcoxon signed rank test compared the results of tips only for restrained and unrestrained cases.

## Results

Experimentally, the center of gravity of the empty wheelchair was found to be centered from side to side, 0.147 m ahead of the rear wheel axis and 0.449 m above the ground. The position of the center of gravity was used to calculate the inertia characteristics of the wheelchair model based on the known masses and geometries of the individual chair components. The calculated I_xx_ for the component chair was 10.70 kg m^2^. This compared well with the I_xx_ determined from the experimental results (10.58 ± 2.55 kg m^2^).

**Fig. 3:**
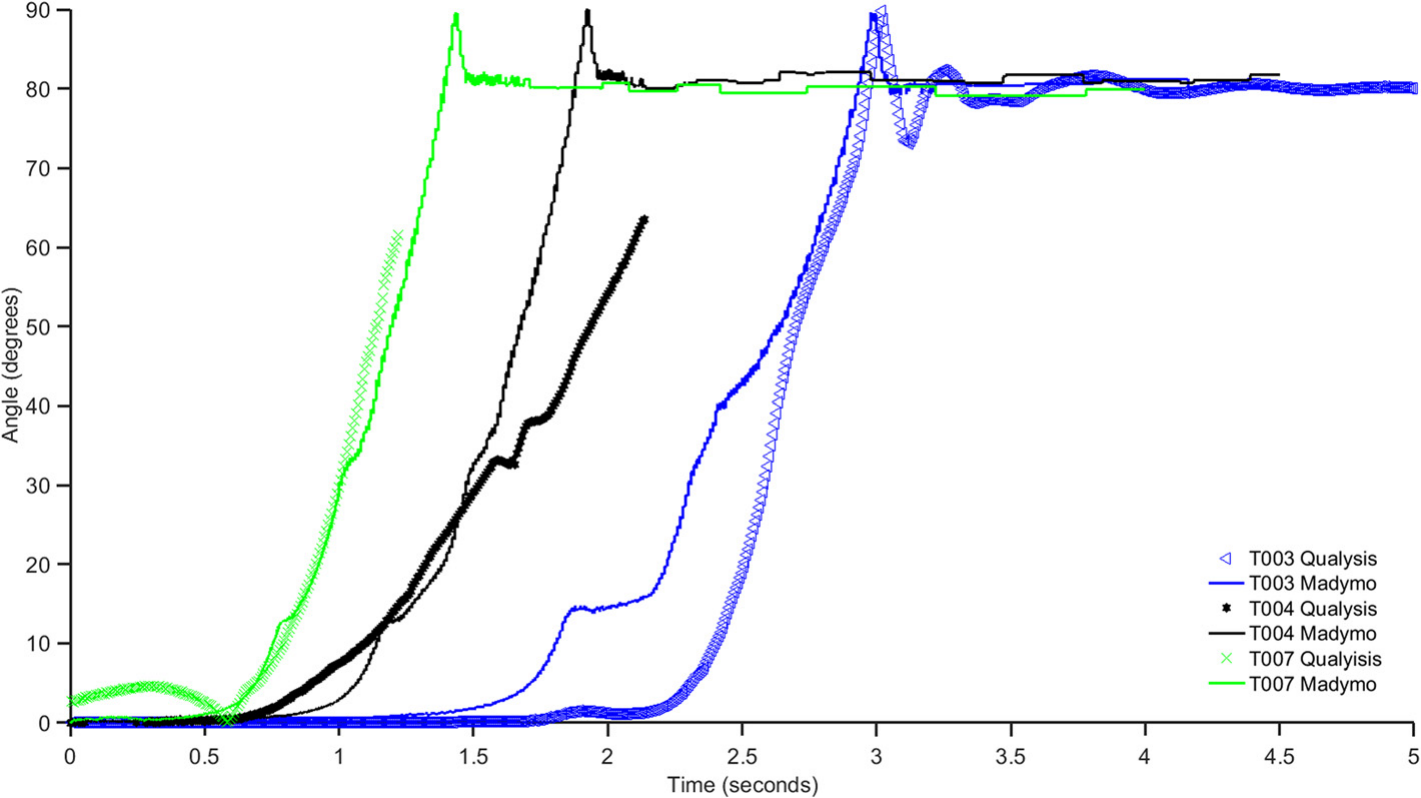
Comparison of simulation (Madymo) and experimental (Qualysis) roll angle for occupied EPW model validation. The slope of the roll data between the experiment and simulation showed good agreement, indicating an accurate moment of inertia in the simulation. Peak roll values are missing from two experiments due to reflective markers being occluded by the dummy arm during tipping. Curbs heights were 0.3 m for all tests. Driving speed and angle varied between tests; T003 - speed = 3.3 m/s, angle = 39.5°, T004 – speed = 1.99 m/s, angle = 20.12°, T007 – speed = 2.35 m/s, angle = 23.12°

Of the eight physical tests with a dummy in the wheelchair, two contained significant yaw during curb interaction due to remote driver error which made the approach angle change as the chair went over the curb. These tests were excluded from the simulation comparison. Three of the remaining tests yielded similar parameter values, therefore, one of these three were selected for simulation. All experimentally-matched simulations resulted in qualitative outcomes (tip, rider, fall, upright) that corresponded to the physical test outcomes. Comparing the motion capture and model data showed consistent roll behaviors between the simulations and experiments that verified the Madymo model dynamic characteristics (Fig. 3).

In the simulations, with a seat belt present, the EPW outright tipped in 16 of the 27 scenarios; 15 were clear tips, however the 1 mph (0.4470 m/s) test at 0.4 m and 60° orientation nearly recovered after becoming stuck on the curb edge (Table 1). With the exception of this test, the same parameter combinations of height and orientation that caused tips at 1 MPH, also resulted in tips at 2 and 3 MPH. Multinomial logit regression showed approach angle was the most significant factor in the occurrence of a tip (*p* < 0.05) while curb height was also a significant factor in wheelchair tips (*p* < 0.05) (Table 2). A more shallow approach to the curb was more likely to result in a tip. Curb height and seatbelt use correlated with the likelihood of falling from an upright wheelchair when compared with staying upright in the wheelchair. The speed of the EPW did not effect tip or fall risk.

**Table 1:** Simulated HIC results for a restrained and unrestrained dummy models and corresponding driving parameter values. HIC values were significantly greater in unrestrained simulations than restrained models. Several unrestrained rider simulations resulted in HIC values exceeded the 1000 threshold (bolded values) that suggests severe head injury may occur

Speed (m/s)	Height (m)	Angle (deg.)	Restrained HIC_15_	Unrestrained HIC_15_
0.45	0.2	25	23.082	60.717
0.45	0.2	45	No Tip	No Tip
0.45	0.2	60	No Tip	No Tip
0.45	0.3	25	26.844	60.824
0.45	0.3	45	18.689	134.04
0.45	0.3	60	No Tip	267.21 (Fall)
0.45	0.4	25	49.541	57.534
0.45	0.4	45	21.249	**1442.1**
0.45	0.4	60	177.64	**1155.7** (Fall)
0.89	0.2	25	17.993	65.535
0.89	0.2	45	No Tip	80.763
0.89	0.2	60	No Tip	No Tip
0.89	0.3	25	25.716	102.83
0.89	0.3	45	16.894	441.66
0.89	0.3	60	No Tip	599.77 (Fall)
0.89	0.4	25	30.666	173.71
0.89	0.4	45	22.644	985.8
0.89	0.4	60	No Tip	21.328 (Fall)
1.34	0.2	25	17.539	290.85
1.34	0.2	45	No Tip	No Tip
1.34	0.2	60	No Tip	No Tip
1.34	0.3	25	29.299	354.44
1.34	0.3	45	17.819	**1463.2**
1.34	0.3	60	No Tip	No Tip
1.34	0.4	25	35.227	**2141.7**
1.34	0.4	45	20.794	**1434.0**
1.34	0.4	60	No Tip	555.65 (Fall)

Bolded values are those exceeding the 1000 threshold, which are thought to predict severe head injury

**Table 2:** Multinomial logit regression showing curb height and approach angle significantly affect the risk of tip

	Multinomial logit estimates^a^			Change in predicted probability (belted)^b^			Change in predicted probability (unbelted)^b^		
Independent variables	Fall vs Upright	Tip vs Upright	Fall vs Tip	Fall	Tip	Upright	Fall	Tip	Upright
Speed (m/s)	−7.96 (4.74)	−0.20 (2.82)	−7.76 (5.38)	0	−0.004	0.013	−0.075	0.075	0.000
Curb Height (m)	84.55** (38.03)	90.83** (38.01)	−6.29 (36.48)	0	0.999	−0.999	0.001	0.896	−0.897
Approach Angle (°)	0.03 (0.25)	−0.89** (0.32)	0.92*** (0.31)	0	−1.00	1.00	0.818	−1.00	0.182
Belted	−17.13** (6.89)	−4.48 (3.67)	−12.66* (6.69)						

Curb height and restraint use are the most significant factors in falls. Driving speed did not affect tip or fall risk. ^a^The values are multinomial logit parameters, standard errors are in brackets. ^b^Change in predicted probability, a positive value means the event is more likely to occur as the parameter value increases, a negative value means the event is more likely to occur as the parameter value decreases (*p = 0.058, **p < 0.05, ***p < 0.005)

In unrestrained simulations, the absence of a restraint allowed for the dummy model to fall forward from the wheelchair - this did not occur when a restraint was present (Table 1). Noting both tip and fall injury mechanisms, curb height was the most significant predictor of staying upright (*p* < 0.05) while chair orientation had a variable effect. All six rider-falls occurred when the wheelchair was oriented at 60° to the curb edge and the passenger was unrestrained; however, tips occurred similarly to the restrained passengers and were more common at low approach angles. In all conditions, restraining the user did not increase the risk of wheelchair tip compared with unrestrained users.

**Fig. 4:**
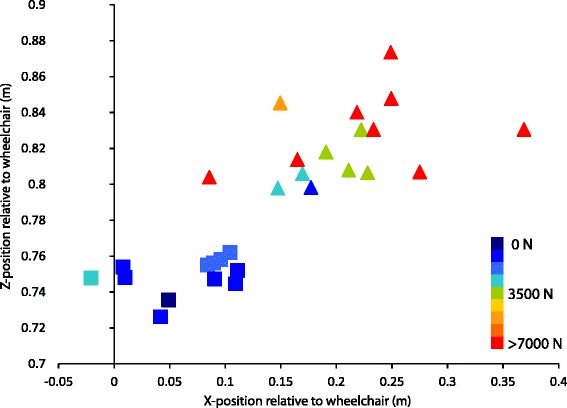
Impact force as a function of impact position for wheelchair tips. Colors represent increasing contact force. All restrained passenger simulations showed contact forces below 3000 N. Unrestrained passengers showed a broad range of impact forces with most simulations exceeding 7000 N. There was a significant relationship between impact position and increased impact force. In the restrained simulations (blocks) the head position is tightly clustered, whereas the unrestrained occupants (triangles) show greater variation in head impact locations relative to the wheelchair. Positive x value indicates the rider moving forward relative to the chair and positive z values indicate the rider moving upward normal to the seat plane

There was a difference between the impact locations of restrained riders and unrestrained wheelchair users with unrestrained users travelling further from the wheelchair and showing greater variation in impact location (Fig. 4). The mean head impact positions along the Z axis (rising from the chair seat) were 0.75 SD 0.01 m for restrained users, and 0.82 SD 0.02 m for unrestrained users. The X axis (forward from the chair seat) component had means of 0.06 SD 0.05 m for restrained users, as opposed to 0.21 SD 0.06 m for the unrestrained users. Users that fell from their chairs while the chair remained upright had the greatest variability in impact position from −1.38 to 2.07 m in the x-direction and −0.10 to 0.27 m in the y-direction (side to side chair position).

Unrestrained users had significantly greater head impact severities than restrained users for all simulations (HIC = 610 ± 634 versus HIC = 29 ± 38, *p* = 0.00013). Several tip events resulted in HICs above 1000 (severe head injury) in unrestrained users while all head impacts in restrained users were mild (<200). In wheelchair tips, there was a strong correlation between head impact position and impact force, with impacts occurring closer to the chair having lower impact forces (Fig. 4). Unrestrained users traveled further from the wheelchair during a tip or fall and had greater average head impact forces (6181 ± 2372 N) than restrained users (1336 ± 827 N) (*p* = 0.00053). The position of the head in the Z-direction (rising from the chair) was strongly predictive of higher impact forces (R^2^ = 0.712, *p* = 0.0002). However, there was no statistical correlation between impact position and HIC magnitude.

## Discussion

EPW’s provide mobility for some of the most vulnerable individuals, and it is important that the risks for injury resulting from tips and falls be quantified and made clear. Wheelchair falls and tips occur frequently and present health care systems with serious and potentially avoidable cases. Recent studies of EPW injury dynamics have yet to quantify the criteria required for effective injury prevention technologies. Having knowledge of the position of the head relative to the wheelchair during impact and the associated forces on the body is instrumental in devising injury prevention or injury mitigation solutions that are wheelchair mounted. The interactions between a passenger and their wheelchair during a tip or fall are complex and require systematic exploration. Computational analysis provides an additional tool to quantify wheelchair fall dynamics in a controlled, systematic way. Quantifying fall dynamics will provide critical design constraints to improve wheelchair safety and to develop protective technologies to prevent tips, falls and injuries.

Computational analyses systematically explore and quantify fall dynamics without the risk to human subjects or the limitations of experiments but themselves are limited by the accuracy of the model assumptions. Models of human injury are limited by their assumptions about the physical characteristics of the body. Using Madymo as the modeling software provided a library of validated human and dummy models to ensure biofidelity [11]. However, the use of dummy models is a limitation of this study because the dummies are passive and provide no active response to tips and falls. While the simulations offer limited insight into the impact characteristics of a rider able to counteract or brace their fall, the study results can be treated as a worst-case scenario. The simulations provide accurate representations of users who are physically unable to react and brace themselves, which would be many power wheelchair users. Additionally, because of the rapid nature of most tips and falls, in many of the simulated falls, the user impacted the ground faster than the perception/reaction time (0.51 s) of the average, healthy individual [15]. This is evident in an observational study of wheelchair fall injuries that found the majority of injuries were traumatic brain injuries and concussions (40 %) and that there were few (10 %) upper extremity injuries [5]. Developing a rigid body model of the power wheelchair required parallel experiments to validate the computational simulations. The wheelchair roll behaviours were well matched between physical and simulated tests (Fig. 2) demonstrating the ability of the simulations to accurately reconstruct experimental tests. The obstacles, orientations and speeds simulated in this study may differ from the ideal environment and are a limitation of this study. Curb heights used in this study (0.2 - 0.4 m) are higher than standard curbs (0.15 m); however, the prevalence of wheelchair tips and falls suggest riders may encounter more severe obstacles than design standards allow. In order to study the dynamics of EPW tips and rider-falls it was necessary to construct environments that would result in falls. The driving speeds studied here represented the range of speeds available on the chair model tested (Ranger Express). The use of a single, rear-wheel drive wheelchair for the experiments may limit the broader application of the results to mid and front wheel drive wheelchairs. However, the impact forces and head injury risk resulting from tips and falls studied here provide some of the first quantitative data on wheelchair fall dynamics and provide important data for designing fall protection systems for these wheelchairs.

HIC value was used as a proxy measure of head injury in this simulation. Although HIC values are well correlated with head injury severity [16] they do not include rotational accelerations that may contribute to mild traumatic brain injuries such as concussions [17]. However, the impact force and HIC values plotted on an impact location map provide important design criteria for reducing impact severity in wheelchair falls.

The results of the fall simulations presented here are consistent with the few prior studies of wheelchair falls. Driving speed had no significant effect on tip or rider-fall risk or impact severity in our study. This agrees with a previous study of power wheelchair falls that found no statistical significance with EPW tip or fall risk, given similar speeds and identical orientation [3]. Additionally, the idea that common obstacle heights can be transversed at 45°, without resulting in a tip is further supported [3]. However, in contrast to the previous study, which found no wheelchair tips occurred in the controlled study environment, we found that power wheelchairs did tip in conditions only slightly beyond current North American accessibility design standards of 100–225 mm curb height [18]. The results showed that approach angle had the greatest effect on tip risk with shallower approaches being more likely to result in a tip. These shallow approach angles may occur with inadvertent drops such as when attempting to avoid obstacles. Separating sidewalks from curbs may help to avoid shallow approach angles to curbs. If curbs must be navigated, this study showed that driving directly off the curb decreased the tip risk compared with a more angled approach; however, unrestrained riders may fall from the wheelchair if curb heights are above design standards. Integrating the results of this study into wheelchair user training [19] should help to prevent tip and fall events.

Our study found that restrained users had much lower impact forces and HIC values than unrestrained riders. This finding agrees with a previous experimental study on wheelchair falls that found less severe impacts in riders restrained with a lap belt compared with those restrained with lap and shoulder belts [20]. When the posture of the body changes during the tip or fall, the impact is affected - the dynamics and location of limbs, and shoulders can influence the resulting head trauma [21]. Sideways falls, like those simulated in the current study, are most common in power wheelchair users and have the highest association with the need for medical treatment [4].

Unrestrained riders were shown to be significantly more prone to injury than their restrained counterparts. This agrees well with previous work studying EPW falls in an outdoor environment [3]. In a controlled study of EPW tips and falls for normal curb heights and unrestrained passengers, HIC measurements neared 1000, [22]. This agrees well with the current findings that had an average HIC of 835 for tips and falls of unrestrained passengers at a curb height of 0.2 m. Importantly, when comparing results between studies it must be noted that variations in EPW makes and models may also affect the tip dynamics and subsequent injuries as a result of variations in the dynamic characteristics of the wheelchair [23].

The HIC magnitude of frontal and side impacts to the head was largely divided in this study - forward falls from the wheelchair resulted in HIC values several times greater than tip injuries. However, the body’s reaction to frontal cranial impacts is not entirely the same as side impacts [24, 25]. A forward fall from a wheelchair restricts the lateral bending of the neck, and the head first impact supports the full mass of the body, which is not the case in side impacts. The high impact peak forces may be a result of the stiff material (concrete) assigned to the impact surface. In addition, the distribution of contact that occurs with a deforming body such as the head is not specifically accounted for in MADYMO simulations and may require further material testing to improve the accuracy of the contact force definitions and resulting head acceleration [26]. However, computational analyses have proven effective for modeling fall behavior and head injury [7–10, 27] and are valuable for assessing the relative effectiveness of protective materials in reducing the severity of fall impacts [10].

Currently, a HIC of 1000 is considered the benchmark for serious head injury - greater than 16 % of the population will incur a life threatening injury with a HIC exceeding 1000 [28, 29]. However, it has been suggested that side impacts to the head with a HIC of 200 can cause severe injury [28, 29]. Therefore, forward falls from the EPW must be treated differently than tips when attempting to protect the rider due to changes in HIC. Forward falls provide a greater challenge for injury mitigation systems, as there was much greater variability in the impact location. However, forward falls only occurred in unrestrained passengers, therefore a seat belt appears to be an effect method for reducing fall and injury risk.

Based on the results of this study and others [3, 20] it would appear that seat belt use should be recommended for all EPW users; however, there are costs and risks associated with seat belts that must also be considered. None of the simulated scenarios resulted in a tipped restrained passenger while the unrestrained person remained upright. Not only did unrestrained scenarios result in more serious HIC measurements, but the variance in the location of impact was greater. This makes it more difficult to design a fall protection solution compared to a restrained passenger. While seat belts provide no observable downside for fall protection, there are usage barriers, complications from use, and stigmas on belt discomfort and appearance that prevent unanimous acceptance [30]. Belt use in wheelchair users can be fatal if the rider slips under the belt resulting in asphyxiation [31, 32]. Belt use for those with spinal cord injuries can lead to reduced circulation and/or pressure sores in the presence of an over-tensioned belt [4]. Oppositely, a lack of belt tension can provide little or no assistance during falls. Importantly a seat belt used for safety can become a physical restraint for riders who are unable to open the latch independently. This can result in significant stress for patients and care providers and is considered unethical and potentially illegal [30, 33]. Practical implementations of restraint systems need to address these issues so that the entire rider community benefits from the potential for reduced or avoided head trauma.

Fall dynamics has been extensively studied in several populations from pediatric to seniors’ falls [27, 34–40]. It is well accepted that understanding and quantifying fall mechanics provides critical insights into fall prevention strategies and injury mitigation approaches such as hip protectors [36, 41, 42], helmets [43] and compliant flooring [44, 45]. The same focus and systematic study has not been applied to understanding wheelchair fall dynamics and therefore injury prevention strategies are not as well established. The dynamic study conducted here showed impact forces and HIC values that correspond to severe head injuries are possible in tip and falls of unrestrained wheelchair users similar to unbraced falls from standing height [46]. Modifying wheelchair designs to limit tips and falls in a range of environments, improving the accessibility of restraint systems, and providing impact mitigating mechanisms such as padded headrests, crash mats or airbags may all have potential in reducing impact forces and resulting head injury risk during wheelchair falls.

## Conclusions

The safety of EPW riders can be addressed to minimize fall occurrences and reduce impact forces. Sideways tips and forward falls from wheelchairs were most sensitive to curb height and approach angle but were not affected by driving speed. Sideways tips and falls resulted in impact forces that could result in concussions or traumatic brain injury and require better injury prevention strategies. While it remains possible for restrained passengers to suffer life-threatening trauma, the risk for unrestrained passengers is much greater due to the possibility of forward falls and general increases in the HIC. However, seatbelt use is currently limited by ethical and legal definitions of restraints. Reductions in any occurrence of injury as a result of wheelchair tips or falls would reduce stress on health care resources both directly through ER visits and indirectly through long-term rehabilitation.
